# Effectiveness of a Mobile Phone Messaging–Based Message Framing Intervention for Improving Maternal Health Service Uptake and Newborn Care Practice in Rural Jimma Zone, Ethiopia: Protocol for a Cluster Randomized Controlled Trial

**DOI:** 10.2196/52395

**Published:** 2024-07-23

**Authors:** Gebeyehu Bulcha, Hordofa Gutema Abdissa, Josef Noll, Demisew Amenu Sori, Zewdie Birhanu Koricha

**Affiliations:** 1 Department of Health, Behavior, and Society Faculty of Public Health, Institutes of Health Jimma University Jimma Ethiopia; 2 Department of Technology Systems University of Oslo Oslo Norway; 3 Department of Obstetrics and Gynaecology Faculty of Medicine, Institutes of Health Jimma University Jimma Ethiopia

**Keywords:** message framing, mHealth, digital health, SMS, maternal health, newborn health, cluster randomized controlled trials, RCT, Ethiopia, mobile phone, effectiveness, SMS-based interventions, text messaging, maternal, newborn care practice, randomized, controlled trial, controlled trials, mobile phone messaging, phone-based intervention

## Abstract

**Background:**

Ethiopia has high rates of maternal and neonatal mortality. In 2019 and 2020, the maternal and newborn mortality rates were estimated at 412 per 1,000,000 births and 30 per 10,000 births, respectively. While mobile health interventions to improve maternal and neonatal health management have shown promising results, there are still insufficient scientific studies to assess the effectiveness of mobile phone messaging–based message framing for maternal and newborn health.

**Objective:**

This research aims to examine the effectiveness of mobile phone messaging–based message framing for improving the use of maternal and newborn health services in the Jimma Zone, Ethiopia.

**Methods:**

A 3-arm cluster-randomized trial design was used to evaluate the effects of mobile phone–based intervention on maternal and newborn health service usage. The trial arms were (1) gain-framed messages (2) loss-framed messages, and (3) usual care. A total of 21 health posts were randomized, and 588 pregnant women who had a gestational age of 16-20 weeks, irrespective of their antenatal care status, were randomly assigned to the trial arms. The intervention consisted of a series of messages dispatched from the date of enrolment until 6-8 months. The control group received existing care without messages. The primary outcomes were maternal health service usage and newborn care practice, while knowledge, attitude, self-efficacy, iron supplementation, and neonatal and maternal morbidity were secondary outcomes. The outcomes will be analyzed using a generalized linear mixed model and the findings will be reported according to the CONSORT-EHEALTH (Consolidated Standards of Reporting Trials of Electronic and Mobile HEalth Applications and onLine TeleHealth) statement for randomized controlled trials.

**Results:**

Recruitment of participants was conducted and the baseline survey was administered in March 2023. The intervention was rolled out from May 2023 till December 2023. The end-line assessment was conducted in February 2024.

**Conclusions:**

This trial was carried out to understand how mobile phone–based messaging can improve maternal and newborn health service usage. It provides evidence for policy guidelines around mobile health strategies to improve maternal and newborn health.

**Trial Registration:**

Pan African Clinical Trials Registry PACTR202201753436676; https://tinyurl.com/ykhnpc49

**International Registered Report Identifier (IRRID):**

DERR1-10.2196/52395

## Background

Ethiopia has one of the highest maternal and neonatal mortality ratios worldwide, with a maternal mortality ratio of 401 per 100,000 live births and a neonatal mortality rate of 33 per 1000 live births [1]. The risk of death due to complications during pregnancy, labor and delivery, and the postnatal period is believed to be reduced with bundles of antenatal care, institutional delivery, and postnatal care (PNC) [2]. However, in Ethiopia, the proportion of women attending at least 4 antenatal visits, delivered at health facilities, and had early PNC service was 43%, 48%, and 35%, respectively [3], indicating there are missed opportunities for almost half of the women who were not able to seek the recommended antenatal visits, institutional delivery, and PNC in the country.

Innovative strategies are required to reduce maternal mortality by improving the use of antenatal care (ANC) services to achieve Sustainable Development Goal 3 by 2030—reducing maternal mortality to fewer than 70 per 100,000 births. Improving child survival also remains a matter of urgent global support. A clear and accessible strategy to achieve this goal is to take advantage of the potential of simple mobile phones to increase the number of ANC visits for prenatal women [4]. Recently, mobile phones have been introduced to disseminate health information and interventions (eg, mobile health [mHealth] interventions) in low- and middle-income countries (LMICs). mHealth is recognized as an important area in implementing global strategies for women’s and children’s health. Although mHealth interventions are promising, there is insufficient scientific research to assess their effects [5]. As of 2022, the number of mobile phone users, including smartphones and feature phones, was 7.26 billion, representing 91.54% of global mobile phone ownership [6]. mHealth has the potential to improve tens of thousands of lives each year. The ubiquity and penetration of mobile phones provide a unique opportunity to leverage mHealth for maternal and newborn care [7]. According to the International Telecommunication Union, mobile phone coverage has increased to 90% of the world’s population and 80% of the rural population [8].

Digital health, or the use of digital technologies for health, has become a prominent field of practice for using routine and innovative forms of information and communications technology to address health needs and improve health knowledge, including in rural areas of LMICs [9,10]. Mobile technology is creating new opportunities to permit safe, accessible, coordinated, and effective maternal and child health care in these regions [11]. Introducing mHealth as a new strategy for health care delivery is seen as a suitable option for reducing the high rates of maternal and child mortality in low-income countries [12,13]. mHealth, a subset of eHealth, is defined as “the use of mobile wireless technologies for health” [9]. SMS is text messaging following mobile network standards. It has been suggested that mobile phone messaging interventions can improve self-management of diabetes, weight loss, physical activity, quitting smoking, and adherence to medication in antiretroviral therapy [14,15]. The use of SMS has also indicated promising outcomes in the promotion of maternal and child health care services [16-20]. However, to date, few studies have evaluated the effect of SMS-based interventions on maternal and newborn health among pregnant and postpartum women in transitioning countries [16,20]. While evidence for SMS-based interventions is well described, the effect of SMS-based message framing intervention for improving maternal and newborn health is limited.

Message framing is the presentation of information in a way that affects the perception, attitude, or behavior of the person receiving it. It is how a message is worded, structured, or framed to impact the target audience’s perception and persuade them to take action [21,22]. There are 2 primary forms of message framing: gain-framing and loss-framing. Gain-framing highlights the advantages or positive results of a decision or action, while loss-framing focuses on the potential negative consequences or losses that could result. The type of framing used depends on the context and objectives of the message, as it can be used to either encourage or discourage specific actions [23,24]. Scholars have been reporting that gain-framed information is more persuasive for disease prevention behaviors as it encourages risk aversion and thus engages in safe disease prevention behaviors. Conversely, loss-framed information is suggested for disease detection behaviors, as it makes people willing to take risks and engage in relatively risky disease detection behaviors [25,26].

The literature on health message framing is extensive and has shown promising effects on health outcomes, including maternal and child health [27,28]. A study by Divdar et al [28] showed significant improvements in oral health knowledge, attitude, intention, efficacy, and practice among the intervention group as compared to the control group, although no significant difference was observed between gain and loss-framed messages. Both gain and loss-framed messages significantly improved breastfeeding self-efficacy in a study conducted in Iran [29]. Another 3-arm intervention has shown significant improvements in complementary feeding knowledge, attitude, self-efficacy, and practice among interventions, with the loss-framed group showing higher improvement than the gain-framed group [30]. However, Frew et al [31] found is no improvement in maternal influenza immunization in their intervention evaluating message framing. These findings indicate inconsistent results regarding which framing method improves maternal and child health care practices [29-31]. Moreover, the evidence on the impact of message framing on maternal and newborn care practice is still unclear in LMICs and needs further assessment [32]. Therefore, to foster our understanding of framing effects and determine which way of information framing has a strong persuasive effect on maternal and newborn health, we need further confirmation.

Although various studies have reported that digital health interventions are effective in improving health service uptake, including maternal and child health services, none, to our knowledge, have considered message framing as a strategy to persuade, change opinions, and encourage the adoption of new behaviors. The main objective of this study is to evaluate the effects of mobile phone messaging-based gain or loss-framed messages on improving maternal health care usage and newborn care practice in the rural Jimma zone.

## Methods

### Setting

The study is being conducted in Manna, Shebe-Sombo, and Dedo districts of Jimma Zone, Oromia, Ethiopia. The districts were selected from 21 districts in the zone after confirming the unavailability of interventions aiming to improve maternal and newborn health services in each of them. Jimma Zone is characterized by weak health infrastructure, high health workers to the population ratio (1:329), only 9 health facilities offering comprehensive emergency obstetric and newborn care and a high maternal mortality ratio (412 per 100,000 live births).

### Study Design

A 3-arm, cluster-randomized trial design with a 1:1 allocation ratio was used.

### Intervention Components

The participants in the intervention were assigned to 3 arms: gain-framed, loss-framed, and control. The detailed description of each arm is presented as follows:

#### Gain-Framed Message

Women in this arm received an automated gain-framed message. The gain-framed SMS text messages, which were tailored based on individual women’s gestational age (GA), expected delivery time, and postnatal period, were sent from the time of enrolment during ANC until the end of the postnatal period to the established list of participants’ phone numbers. Women in this group also received the existing maternal care provided at the health facility.

#### Loss-Framed Messages

Women in this arm received automated, loss-framed messages. The loss-framed SMS text messages, which were tailored based on individual women’s GA, expected delivery time, and postnatal period, were sent from the time of enrolment during ANC until the end of the postnatal period to the established list of participants’ phone numbers. Women in this group also received the existing maternal care provided at the health facilities.

#### Control Group

Participants in the control group received existing ANC and PNC services provided at health facilities. According to the recent ANC guidelines, pregnant mothers are expected to have 8 ANC and 3 PNC contacts. In addition to these cares, counseling services is recommended on the following topics during ANC visits: (1) lifestyle modification, (2) danger signs and symptoms, (3) birth preparedness and complication readiness, and (4) other issues such as family planning, cord care, bathing, breastfeeding, child immunization, and screening for cervical cancer [33].

### Intervention Packages

The intervention messages and the messaging system, which automatically dispatches these messages, were developed. These messages were developed based on the concept of message framing, with half of them developed in the form of a gain frame—emphasizing the benefits of maternal and newborn health care practices (eg, choosing to put your new baby to the breast in the first hour he or she is born will increase a baby’s protection from many diseases and illnesses). The other half of the messages were developed as a loss frame—emphasizing loss as a result of not using maternal health services and practicing newborn care (eg, failing to put your new baby to the breast in the first hour he or she is born will lower a baby’s protection from many diseases and illnesses). Multimedia Appendix 1 provides more examples of both gain- and loss-framed messages.

The content of the messages was evaluated by mothers and experts before it was used in the intervention. Initially, 120 (60 for each form) English version messages were drafted within the character count of 160, based on the Ethiopian Ministry of Health’s guidelines and the World Health Organization’s recommendations. After discussing with the research team, 32 (16 from each form) messages were discarded. The remaining 88 messages were translated into the Afan Oromo language and used for content evaluation. Mothers evaluated the content of these 88 messages through a card sorting activity, and 10 messages (5 from each form) that were not well understood by mothers were discarded. Following the card sorting activity, 8 experts evaluated the retained 78 messages (39 for each form) using 10 statements. The experts suggested modifications for 14 messages (13 loss-framed and 1 gain-framed), and all of these messages were modified and refined based on the suggestions.

Finally, 39 messages for each message form were retained under 10 thematic areas: nutrition, lifestyle modification, danger signs, birth preparedness, complication readiness, breastfeeding practice, labor, newborn care, immunization, and antenatal and PNC usage. These messages were dispatched using locally developed software that manages the schedule and sends the messages automatically. To facilitate this, a domain, layer 3 VPN, and short code were purchased from Ethio-telecom Company. The software program includes an interface to enter the participant’s name, gestational week, mobile phone number, message type, message category, language option, report generation, and feedback options.

### Eligibility Criteria for Clusters and Participants

Clusters (Kebeles) with good mobile network coverage in 3 of the selected districts were eligible for randomization. Pregnant women with a GA of 16-20 weeks who either owned a mobile phone themselves or had a family member who owned one, lived in the selected cluster for at least 6 months, provided consent to participate in the intervention, and shared their phone number were eligible for the trial. Pregnant mothers who were unable to read mobile phone messages and had plans to relocate from the study cluster during the follow-up period were excluded from the intervention.

### Sample Size Determination

#### Sample Size for the ANC Visit

The method suggested by Hooper et al [34] for cluster randomized trials with repeated cross-sections was used for the calculations. This method requires calculating 2 design effects, which are then used to inflate the sample size for individual randomization to account for the within-period intracluster correlation coefficient (ICC) and the between-period ICC. A sequence of steps has been followed to get the final sample size for the current 3-arm cluster randomized trials with repeated cross-sections. For ANC, we first calculated the individual randomization trial using the following assumptions: P1—the proportion of women attending ANC for more than 4 visits among the control group 23.27% [35], power=80%, 2-sided α=0.025, effect size=15%, and Zα/2 adjusted for multiple comparisons using a Bonferroni correction of 2.24. Individual randomization sample size (n) is calculated as follows:

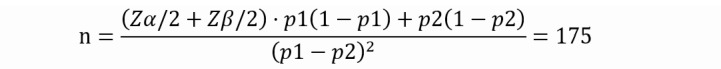



Next, the design effect (*d*_c_) due to cluster randomization was calculated as *d_c_* = 1 + (*m* – 1)*ρ*. Here, the following parameters were considered: the within-period ICC(*p*) was assumed to be 0.003 [36], and the cluster size (m) was assumed to be 28. The calculation yielded a design effect due to cluster randomization *dc* = 1 + (*m* – 1)*ρ* = 1.08. Additionally, the design effect due to repeated assessment was calculated as *d*_r_=(1–*r*^2^), where *r* is the correlation between samples from the same cluster at different times and determined as 
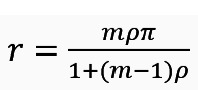
. Based on this, *d*_r_ was calculated using assumptions: within-period ICC=0.003, autocorrelation coefficient (π) of 0.8 (to allow for a 20% decay of the correlation from within to between different periods) [37], and *m*=28 and 
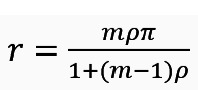
=0.062. Thus, the design effect due to repeated assessment was *d*_r_=(1–*r*^2^)=0.996.

To arrive at the final sample size per arm, the sample size for individual randomization is multiplied by both design effects, resulting in (n×*d*_c_×*d*_r_)=188. Considering a 4% loss to follow-up, the final sample size for each arm is 196 and this gives a total sample size of 588 when ANC was used as the primary outcome of the intervention.

#### Sample Size for Newborn Practice

For newborn care practice, the same procedure described earlier was used. First, the individual randomization sample size was calculated to be 118 based on the following assumptions: P1—the proportion of exclusive breast feeding among the control group of 74.8% [38], power=80%, 2-sided α of 0.025, effect size=15%, and *Z*α/2 adjusted for multiple comparisons using a Bonferroni correction=2.24. Then, the design effect (*d*_c_) due to cluster randomization was calculated as *d_c_* = 1 + (*m* – 1)*ρ* = 1.35 by considering the following parameters: within-period *p* of 0.013 [39] and cluster size assumed to be 28. The design effect due to repeated assessment was calculated as *d*_r_=(1–*r*^2^)=0.953, where the correlation between samples from the same cluster at different times *r*=0.251 by using assumptions: within-period ICC=0.013 [39], autocorrelation coefficient (π) of 0.8 (to allow for a 20% decay of the correlation from within to between different periods) [37], and *m*=28. By inflating the sample size under individual randomization with the 2 design effects as (n×*d*_c_×*d*_r_)=152. Considering a 4% loss to follow-up, the total sample size was 475.

Therefore, the sample calculated for ANC was larger (588) and was the final sample size of the trial to arrive at 21 clusters (7 per arm) and an average of 28 eligible pregnant mothers.

### Recruitment

Following the randomization of the clusters, lists of pregnant women in the randomized kebeles were obtained from the health extension worker’s registration book. Midwives then identified eligible pregnant women using 2 strategies: the last menstruation period (LMP) and the fundal height measurement technique (in the case of an unknown LMP) to determine the GA of mothers. The data collector provided a detailed explanation of the inclusion criteria and overall intervention procedure to eligible mothers by using the information sheet. Eligible pregnant mothers were enrolled in the intervention after obtaining informed consent regarding their willingness to give their phone number for the intervention and their approval to receive text messages to their own or their family’s phone during the intervention period. The recruitment was done after randomly selecting 28 participants from a list of eligible pregnant mothers in each cluster. After recruitment, information, such as the participant’s name, mobile phone number, and GA, was gathered during the baseline assessment. This information was then entered into the software to dispatch the messages according to the GA of those assigned to the intervention arm.

### Randomization, Allocation Concealment, and Blinding

A total of 6 primary health care units, 2 from each district, were randomly selected, and kebeles in these primary health care units were used as units of randomization for the trial. A simple randomization with a 1:1 allocation was used to randomize the clusters (kebeles). After listing 30 kebeles that fulfilled the eligibility criteria, 21 of them were randomly selected for randomization. To assign the clusters to the intervention groups (gain-framed group, loss-framed group, and control group), the following steps were followed. First, the 21 clusters were alphabetically listed and labeled 1 to 21. Next, random numbers were generated using Excel 2019 (Microsoft Corp), and the generated values were copied and pasted as values next to the list of clusters. The list was then sorted in ascending order based on the generated random number. Finally, the first 7 clusters in the sorted list were assigned to the gain-framed group, the next 7 to the loss-framed group, and the last 7 to the control group. After the randomization, eligible pregnant women with a GA of 16-20 weeks were identified from the selected cluster by midwives. The midwives used 2 strategies: the LMP and the fundal height measurement technique in the case of an unknown LMP to determine the GA of mothers (Multimedia Appendix 2).

The allocation process was done by a person who was not involved in the implementation of the trial, and it was masked from the researcher involved in the implementation of the intervention. The interviewers who collected the data were blind to the intervention assignment. It is not possible to blind participants due to the nature of the intervention.

### Outcomes

The primary and secondary outcomes are provided in Textbox 1.

**Primary and secondary outcomes.**

**Primary outcomes**

- Maternal health service usage
- Newborn care practice

**Secondary outcomes**

- Knowledge about maternal health services
- Knowledge of danger signs (during pregnancy, labor and delivery, and the postnatal period)
- Knowledge of newborn care practice
- Knowledge about newborn danger signs
- Attitude toward maternal health services
- Attitude toward newborn care
- Self-efficacy toward maternal health service usage
- Self-efficacy toward newborn care practice
- Iron supplementation
- Maternal morbidity
- Neonatal morbidity

### Operational Definitions

Operational definitions are as follows:

- Maternal health service usage:
  when mothers receive at least 8 ANC follow-up visits, give birth in a health facility, and visit a health facility for PNC follow-up contact from health professionals within 7 days of delivery.
- Newborn care practice:
  this refers to the care provided to the baby from birth to 28 days of age by the mothers, including early initiation of breastfeeding, exclusive breastfeeding, feeding colostrum, thermal care, hygienic cord care, and seeking care for an ill newborn. If a mother feeds colostrum, initiates breastfeeding within 1 hour after birth, feeds a newborn only breast milk, practices thermal and hygienic cord care, and seeks care for her ill newborn, she is considered to be practicing newborn care, and if she misses one of them, she is considered to be not practicing newborn care.
- Knowledge about maternal health services:
  participants were asked to mention the recommended newborn care practice using 16 questions. All the responses were counted, and the higher score indicates a higher knowledge of newborn care practice.
- Knowledge of maternal danger signs:
  this was measured by 7 items related to maternal danger signs during pregnancy, labor & delivery, and the postnatal period such that yes=1 for correct statements and no=0 for incorrect statements. The scores of correct answers were summed, and a higher score was interpreted as a higher level of knowledge of maternal danger signs.
- Knowledge of newborn danger signs:
  this was measured by 12 items related to neonatal danger signs, such that yes=1 for correct statements and no=0 for incorrect statements. The scores of correct answers were summed, and a higher score was interpreted as a higher level of knowledge of newborn danger signs.
- Knowledge of newborn care practice:
  participants were asked to mention the recommended newborn care practice using 7 questions. All the responses were counted, and the higher score indicates a higher knowledge of newborn care practice.
- Attitude toward maternal health services:
  this refers to mothers’ feelings toward maternal care practice. Attitude toward maternal care practice was measured by 10 items on a 5-point Likert scale ranging from 1=strongly disagree to 5=strongly agree. The cumulative scores were used in the analysis once negatively phrased items were reverse-coded. A higher composite score indicated a more favorable attitude toward maternal care practice.
- Attitude toward newborn care practice:
  this refers to mothers’ feelings toward newborn care practices. It was measured by 16 items, which are designed in a 5-point Likert scale format ranging from 1=strongly disagree to 5=strongly agree. The sum score was used in the analysis after reverse coding the negatively worded items. The higher composite score was interpreted as a favorable attitude toward newborn care practice.
- Self-efficacy to practice maternal health service usage:
  self-efficacy in practicing maternal care is an individual belief in mothers’ ability to perform maternal care practices. It was measured by 10 items designed on a 5-point Likert scale ranging from 1=strongly disagree to 5=strongly agree. Once negatively worded items were reverse-coded, the sum score was used in the analysis. A score with a high value indicated greater self-efficacy in practicing maternal care.
- Self-efficacy to practice maternal and newborn care:
  this refers to an individual belief in one’s ability to perform maternal and newborn care practice. It was measured by 9 items, which are designed in a 5-point Likert scale format ranging from 1=strongly disagree to 5=strongly agree. The sum score was used in the analysis after reverse coding the negatively worded items. A score with a high value indicates higher self-efficacy toward practicing newborn care.

### Data Collection Procedures and Tools

The baseline survey was conducted in March 2023, while the end-line survey was conducted in February, 2024. Trained interviewers collected data for both the baseline and end-line surveys through face-to-face interviews using a structured questionnaire. The questionnaire addressed various domains, including sociodemographic factors, socioeconomic factors, obstetric history, maternal health service usage, newborn care practice, knowledge about maternal health services, knowledge about maternal danger signs, knowledge about newborn danger signs, knowledge about newborn care practices, attitude toward maternal health services, attitude toward newborn care practice, self-efficacy toward maternal health services, self-efficacy toward newborn care practice, iron supplementation, and maternal and neonatal morbidity. Participants who were lost to follow-up were recorded, along with their reasons for discontinuation and the outcome of their pregnancy. The data were checked for inconsistencies and errors during the data collection period by dedicated supervisors and submitted to the central data management unit.

A total of 10 data collectors and 2 supervisors were recruited during each data collection period. A 2-day training was given for data collectors and supervisors on the questionnaire content, measurements, ethical issues, and how to use the ODK app (during end-line assessment), and they were kept uninformed about cluster allocation.

### Data Management

The collected data during the baseline and end-line was checked for completeness at the time of data collection by supervisors, and questionnaires with missing items were communicated with data collectors for correction. Double entry was done for baseline data to identify and manage mistakes during the data entry, while the end-line data were retrieved from KoBo Toolbox. Data cleaning was done to identify the duplicate records and missing and outlier values; to verify that the values were in the proper format and expected range; and to correct the errors. Only the study team has access to the intervention data sets. Information about clusters and participants was not shared with any third party, both during and after the intervention. Moreover, all personal identifiers were removed from the database, and paper-based filled questionnaires were stored in a locked cabinet. Data were stored in a database on a password-protected computer, and only the research team could access it.

### Data Analysis Plan

Double-data entry was accomplished using Epi-data (version 4.6, released in 2020; the EpiData Association) and exported to the SPSS (version 25; IBM Corp) for analysis. The report of this study will be made according to the CONSORT-EHEALTH (Consolidated Standards of Reporting Trials of Electronic and Mobile HEalth Applications and onLine TeleHealth) standards for reporting randomized controlled trials. Exploratory subgroup analysis was done to identify whether the effects of intervention vary with sociodemographic characteristics, socioeconomic characteristics, obstetric history, and psychological factors. To assess the characteristics of the study population through the lenses of different variables, selected data were tabulated by study arms; this helped us to identify variations and similarities among study arms. The summary statistics used for categorical variables were frequencies and percentages. For continuous variables, the mean and standard deviation will be presented.

To observe the effectiveness of mobile phone messaging-based message framing interventions for improving maternal and newborn health service usage, we will use an intention-to-treat approach where all randomized participants will be included in the statistical analysis, regardless of whether they received the intervention or not. A generalized linear mixed model will be used to adjust for both the clustering effect and the participant-level intracluster correction. The outcome variables are assumed to have a multivariable normal distribution. The odds ratio will be used to measure the effectiveness of the intervention on the outcome variable with a 95% CI.

### Ethical Considerations

Ethical approval was received from the Jimma University Institutional Review Board (ref no JUIH/IRB/358/23). Participating in this trial was completely voluntary, and women can opt out of receiving the messages by sending “stop” to the domain. Informed consent was obtained for taking part in the intervention, baseline, and end-line assessments. The interviewer read the contents of the informed consent form and outlined the purpose of the study, the institutions and researchers involved, the expectations of women, and the risks and benefits associated with the trial using the local language. Women also get an explanation about their rights as participants and have their questions answered before enrollment.

Women’s phone numbers were kept encrypted, and no one could access them without a server technician and researcher. Codes were assigned to each enrolled woman to ensure confidentiality; mobile numbers were entered into the dashboard anonymously. Data were shared with the research team after removing participants’ names. No compensation was provided to participants for taking part in this trial.

### Monitoring

Monthly monitoring of intervention implementation was conducted by collecting data through face-to-face interviews using a predefined questionnaire. Participants were asked the number of maternal and newborn information they have received through SMS, the number of messages read, the service they have used following the received message, and any pregnancy outcomes. They were also asked about any barriers that they have faced to receive the messages and the usage of services after receiving messages. Moreover, the functionality and stability of the messaging system were monitored continuously for the duration of the project. Data about the SMSs being sent and delivered were also automatically recorded by the messaging system and checked weekly.

## Results

The recruitment of 588 intervention participants and the baseline survey were carried out in March 2023. The intervention was rolled out starting in May 2023 and ending in December 2023. The end-line assessments were conducted in February 2024. We are working on data management, and we expect the analysis to be completed by May 30, 2024. The publication of the results is anticipated in August, 2024.

## Discussion

### Major Findings

This study aims to evaluate the effectiveness of mobile phone messaging–based message framing interventions for improving maternal and newborn health practices. We hypothesize that increased usage of maternal and newborn care services in the intervention group exposed to mobile-based message framing (gain-framed and loss-framed messages) was comparable to that in the control arm. This trial is designed with the assumption of achieving improvements in key indicators such as 8-time antenatal care visits, increased health facility deliveries, increased postpartum attendance, and improved newborn care practice. Previous studies indicate that mobile phone interventions during pregnancy have a positive effect on maternal health outcomes. For instance, text messaging interventions have shown potential for informing, motivating, and reminding pregnant and postpartum women about timely health care usage, which leads to positive maternal and child health outcomes [40-43].

Our intervention focuses on message framing for the early enrollment of pregnant women on the continuum of care. Pregnant mothers with a GA of 16-20 weeks are preferred based on the World Health Organization’s recommendations for early antenatal care initiation. Previous studies evaluating the effectiveness of SMS-based interventions to improve maternal and neonatal health selected pregnant women who had already started antenatal care visits and used health facilities [12,44-46].

### Strengths and Limitations

This study uses a cluster randomized controlled trial (CRCT) design to address shortcomings in prior observational and pseudo-experimental studies. This study attempts to minimize contamination by cluster randomization at the health post level. Many previous studies were observational and pseudo-experimental in nature, which have inherent biases because they lack randomization, which leads to inference problems and difficulty in group comparability. This suggests that there is a methodological gap that requires generating more reliable information by using stronger designs such as CRCTs [47-50].

To reduce the risk of incomplete or inconsistent data, supervision of field-based data collection is sustained throughout the entire data collection period. The use of mediation analysis examines the intricate relationship between participants’ adherence to the mobile messaging intervention and the observed changes in maternal health service uptake and newborn care practices, offering a comprehensive understanding of its impact. Subgroup analysis is performed to investigate differences in results among various clusters, considering unique contextual factors that may influence the intervention’s impact on clusters.

The intervention could have some limitations that should be acknowledged. One limitation is that the study included only pregnant mothers who have mobile phones. Consequently, the findings may not be generalized to all pregnant mothers. Focusing on pregnant mothers who own mobile phones is the drawback of this study, as it has the potential to marginalize those who do not have mobile phones. Another limitation of the study is the failure to assess the cost-effectiveness of the interventions, which is essential for informing resource allocation decisions and promoting their adoption in low-resource settings.

Future research directions could involve scaling up the mobile phone messaging intervention to reach a larger population or exploring additional strategies to complement its effects. Integrating mobile phone messaging interventions within broader health care frameworks could enhance their effectiveness in promoting maternal health service uptake. To ensure the sustainability and long-term impact of mobile phone messaging interventions, it is crucial to integrate them into existing health care systems. This could involve collaborating with local health authorities to develop policies and guidelines for the use of mobile health interventions in maternal and newborn care. Conducting long-term follow-up studies can provide valuable insights into the sustained impact of mobile phone messaging interventions on maternal health service uptake and newborn care practices. Assessing the cost-effectiveness of mobile phone messaging interventions is essential for informing resource allocation decisions and promoting their adoption in low-resource settings.

### Dissemination Plan

The dissemination plan involves publishing results in peer-reviewed journals, presenting the findings at conferences, engaging with local health authorities, and collaborating with community organizations for implementation.

### Conclusions

To the best of our knowledge, this study is the first CRCT to evaluate the effectiveness of a mobile phone messaging-based message framing intervention in improving maternal health service uptake and newborn care practice in Ethiopia. The evidence from this study will be crucial for policy makers to scale up as a strategy to reduce maternal and neonatal mortality rates.

## Appendix

Multimedia Appendix 1 Example gain and loss message types from ten thematic areas.

Multimedia Appendix 2 The flow of participants through each stage of the randomized trial, including enrollment, allocation, follow-up, and analysis.

## Abbreviations

ANC: antenatal care
CONSORT-EHEALTH: Consolidated Standards of Reporting Trials of Electronic and Mobile HEalth Applications and onLine TeleHealth
CRCT: cluster randomized controlled trial
GA: gestational age
ICC: intracluster correlation coefficient
LMIC: low- and middle-income country
LMP: last menstruation period
mHealth: mobile health
PNC: postnatal care
