# Supplementary material for: Effectiveness of a Mobile Phone Messaging–Based Message Framing Intervention for Improving Maternal Health Service Uptake and Newborn Care Practice in Rural Jimma Zone, Ethiopia: Protocol for a Cluster Randomized Controlled Trial
Source: JMIR Res Protoc. 2024 Jul 23;13:e52395. doi: 10.2196/52395 (PMC11303884; doi:10.2196/52395)
Supplement: Multimedia Appendix 1 [file resprot_v13i1e52395_app1.docx]

**Multimedia Appendix 1:** **Example gain and loss message type from ten thematic area**

| **Message category** | **Gain Framed Messages** | **Loss Framed Messages** |
| --- | --- | --- |
| **ANC schedule** | For normal pregnancy, experts recommend 8 antenatal contacts. If you follow recommended and regular antenatal care visits, you and your baby will be best protected | For normal pregnancy, experts recommend 8 antenatal contacts. If you do not follow recommended and regular, antenatal care visits, you and your baby will not be best protected |
| **Nutrition** | Eating a balanced diet with high of whole grains, vegetables, fruits, low fat dairy, and lean protein to remains healthy and have a healthy baby | If you don’t eat a balanced diet with high of whole grains, vegetables, fruits, low fat dairy, and lean protein, your chance of illness and having ill baby will increase |
| **Lifestyle modification** | Avoiding unwashed vegetables and fruit, raw milk, processed/packed food for your health and your baby | Consuming unwashed vegetables and fruit, raw milk, processed/packed food can make you ill |
| **Danger sign** | If you contact health worker when you notice decreased or lost fetal movement, you can learn what is abnormal and get early treatment | If you don’t contact health worker when you notice decreased or lost fetal movement, you cannot learn what is abnormal and do not get early treatment |
| **Birth preparedness** | By having 2 clothes ready to 1 to dry and 1 to wrap after birth, you will take advantage of keeping your baby warm so that he/she will be healthy | By failing to have 2 clothes ready to 1 to dry and 1 to wrap after birth, you won’t take advantage of keeping your baby warm so that he/she will become ill |
| **Breastfeeding practices** | Choosing to put your new baby to the breast in the first hour he/she is born will in increases a baby’s protection from many diseases and illnesses | Failing to put your new baby to the breast in the first hour he/she born will lowers a baby’s protection from many diseases and illnesses |
| **Newborn care** | If you sponge the cord with clean water, leave it uncovered to dry and put nothing on it, you will prevent cord infection which make your baby very ill | If you don’t sponge the cord with clean water, covered it and put anything on it, you will not prevent cord infection which make your baby very ill |
| **Child Immunization** | By vaccinating your child according to a schedule, you will take advantage of your child protection against contracting many diseases | By not vaccinating your child according to a schedule, you will fail to protect your child against contracting many diseases |
| **Laboring** | Strong contractions and jelly-like substance on underwear are a sign of labor. By going to health facility on time, you will have better pregnancy outcome | Strong contractions and jelly-like substance on underwear are a sign of labor. Failing to go to health facility on time, may lead to bad pregnancy outcome |
| **PNC** | One of the greatest advantages of going to health facility when there are severe headaches, blurred vision and high fever after child birth is saving oneself | One of the greatest disadvantages of not going to health facility when there is severe headaches, blurred vision and high fever after child birth is failing to saving oneself |
