# Supplementary material for: Effectiveness of a Mobile Phone Messaging–Based Message Framing Intervention for Improving Maternal Health Service Uptake and Newborn Care Practice in Rural Jimma Zone, Ethiopia: Protocol for a Cluster Randomized Controlled Trial
Source: JMIR Res Protoc. 2024 Jul 23;13:e52395. doi: 10.2196/52395 (PMC11303884; doi:10.2196/52395)
Supplement: Multimedia Appendix 2 [file resprot_v13i1e52395_app2.docx]

Assessed for eligibility (n=37 clusters)

Excluded (n= 16)

♦  Not meeting inclusion criteria (n= 7 )

♦  Declined to participate (n= 0 )

♦  Other reasons (n=9 )

Analysed

Clusters (n=7)

Mothers ( n=196)

Follow-up for eight months

Allocated to gain framed intervention (n=7 clusters)

 Mothers who Receives allocated intervention (n= 196 )

Follow-up for eight months

Allocated to loss framed intervention (n= 7 clusters)

Mothers who receives allocated intervention (n= 196)

Analysed

Clusters (n=7)

Mothers ( n=196)

Allocation

Analysis

Follow-Up

Randomized (n= 21)

Enrolment

Allocated to usual care intervention (n=7 clusters)

Received allocated intervention (n=196)

Follow-up for eight months

Analysed

Clusters (n=7)

Mothers ( n=196)

**Multimedia Appendix 2:** The flow of participants through each stage of the randomized trial, including enrollment, allocation, follow-up, and analysis.
